# Aperiodic EEG Predicts Variability of Visual Temporal Processing

**DOI:** 10.1523/JNEUROSCI.2308-23.2024

**Published:** 2024-08-21

**Authors:** Michele Deodato, David Melcher

**Affiliations:** ^1^ Psychology Program, Division of Science, New York University Abu Dhabi, Abu Dhabi, United Arab Emirates; ^2^ Center for Brain and Health, NYUAD Research Institute, New York University Abu Dhabi, Abu Dhabi, United Arab Emirates

**Keywords:** aperiodic EEG, neural noise, temporal processing, visual perception

## Abstract

The human brain exhibits both oscillatory and aperiodic, or 1/*f*, activity. Although a large body of research has focused on the relationship between brain rhythms and sensory processes, aperiodic activity has often been overlooked as functionally irrelevant. Prompted by recent findings linking aperiodic activity to the balance between neural excitation and inhibition, we investigated its effects on the temporal resolution of perception. We recorded electroencephalography (EEG) from participants (both sexes) during the resting state and a task in which they detected the presence of two flashes separated by variable interstimulus intervals. Two-flash discrimination accuracy typically follows a sigmoid function whose steepness reflects perceptual variability or inconsistent integration/segregation of the stimuli. We found that individual differences in the steepness of the psychometric function correlated with EEG aperiodic exponents over posterior scalp sites. In other words, participants with flatter EEG spectra (i.e., greater neural excitation) exhibited increased sensory noise, resulting in shallower psychometric curves. Our finding suggests that aperiodic EEG is linked to sensory integration processes usually attributed to the rhythmic inhibition of neural oscillations. Overall, this correspondence between aperiodic neural excitation and behavioral measures of sensory noise provides a more comprehensive explanation of the relationship between brain activity and sensory integration and represents an important extension to theories of how the brain samples sensory input over time.

## Significance Statement

Neural oscillations are fundamental for temporal organization and communication of information in the brain, while irregular activity has been suggested to disrupt these processes. We investigated the impact of aperiodic electrophysiological activity on a visual integration task known to depend on rhythmic brain organization. Crucially, individuals exhibiting higher 1/*f* electrophysiological noise showed increased uncertainty or perceptual noise in temporal perception, highlighting how irregular neural activity affects tasks relying on periodic neural organization. These findings significantly advance our understanding of aperiodic electroencephalography and its implications for perception, shedding light on its intricate interplay with various neural processes.

## Introduction

Since Berger's first observation of the alpha rhythm in human electroencephalography (EEG; 1929), the study of brain oscillations and their potential roles in cognition have been a major focus of neuroscience research. Rhythmic patterns of brain activity are typically identified as peaks in the EEG power spectrum, with the alpha rhythm in the 8–12 Hz range being the most prominent. It has been proposed that alpha oscillations act as a sensory sampling mechanism, meaning that the duty cycle of the alpha rhythm (∼100 ms) corresponds to a perceptual snapshot or a temporal integration window (VanRullen and Koch, 2003; Ronconi et al., 2017). In support of this idea, studies have found that the period of alpha oscillations predicts many behavioral measures of temporal processing and integration (for a review, see Samaha and Romei, 2023). From a physiological standpoint, EEG alpha oscillations represent waxing and waning of inhibitory neural activity in the brain (Romei et al., 2008). Thus, sensory parsing could be achieved by regularly pulsed neural inhibition (Mazaheri and Jensen, 2010; Mathewson et al., 2011). However, recent null findings challenge the view that sensory integration may depend solely on oscillatory processes (Ruzzoli et al., 2019; Keitel et al., 2022). Specifically, it has been suggested that internal noise could also affect the temporal organization of perception (Yarrow et al., 2022; Deodato and Melcher, 2023).

Apart from peaks of periodic activity, the EEG power spectrum exhibits a predominant aperiodic component, where power decreases as frequency increases following a power law or 1/*f* pattern, suggesting the presence of irregular or nonrhythmic brain activity (He et al., 2010). Indeed, 1/*f* activity has been observed not only in M/EEG recordings but also in local field potentials and functional magnetic resonance imaging (Manning et al., 2009; Miller et al., 2009; He, 2011, 2014; Ray and Maunsell, 2011; Donoghue et al., 2020).

Recent studies have shown that aperiodic brain activity can provide important insights into brain functioning (Churchill et al., 2016) and disease (Pani et al., 2022). For example, the steepness of the electrophysiological power spectrum, or aperiodic exponent, has been linked to the firing statistics of the underlying neural population and excitation/inhibition ratio (El Boustani et al., 2009; Gao et al., 2017; Lombardi et al., 2017; Wiest et al., 2023). In the aging context, flatter spectra have been associated with asynchronous, more random, firing and neural activity decoupled from brain rhythms (Voytek et al., 2015; Voytek and Knight, 2015; Tran et al., 2020). Reduced aperiodic exponents, or a “flatter” spectrum, have been consistently reported in older individuals compared with young ones (Voytek et al., 2015; Waschke et al., 2017; Cesnaite et al., 2023) and have been linked to processing speed (Ouyang et al., 2020; Pathania et al., 2022) as well as broader domains of cognitive performances (Pei et al., 2023; Euler et al., 2024). In line with these findings, a novel framework proposed that aperiodic exponents could represent asynchronous excitation and “neural noise”, justifying its relationship with oscillations and behavior (Voytek et al., 2015; Voytek and Knight, 2015; Tran et al., 2020). Accordingly, through this paper, we define neural noise as neural spiking decoupled from an organizing neural rhythm and driven instead by a positive imbalance in the baseline excitation/inhibition ratio that is reflected at the meso-/macroscale in decreased steepness of the power spectrum (Voytek and Knight, 2015).

While in a previous study we focused on the relationship between speed of oscillations and visual temporal acuity (Deodato and Melcher, 2023), here, we reanalyzed this EEG dataset to investigate whether aperiodic EEG activity is irrelevant for visual processing or, instead, has functional significance for the temporal resolution of perception. Oscillations and aperiodic activity may represent two sides of neural communication, whose balance directly affects perception and, more generally, cognition. Specifically, we reasoned that alpha oscillations’ pulsed inhibition mechanism could be altered by different levels of excitation/inhibition balance. Greater neural noise and excitation could affect the rhythmic temporal organization of the visual flow by increasing the variability of neural processing latencies (Yarrow et al., 2022) and trial-by-trial response variability (Tran et al., 2020) or weakening oscillatory coupling (Voytek and Knight, 2015). Importantly, at the behavioral level, a noisier or unreliable sensory parsing/organization process should be observable in less reliable psychometric temporal thresholds or shallower psychometric curves (Deodato and Melcher, 2023).

Overall, uncovering the role of the aperiodic component of EEG, in addition to oscillations, for temporal perception might provide an important extension to theories of rhythmic sensory sampling and a more comprehensive account of the relationship between brain activity and temporal integration/segregation, including a possible explanation of null findings.

## Materials and Methods

### Participants

A total of 50 participants (26 females) between 18 and 50 years old (age mean, 21.62 years; SD, 5.33) participated in the experiment and received compensation. Inclusion criteria were normal or corrected to normal vision and English fluency. Data were collected in accordance with the Declaration of Helsinki, and the study protocol was approved by the local ethics committee (New York University Abu Dhabi IRB).

### Experimental design

**Figure 1.**
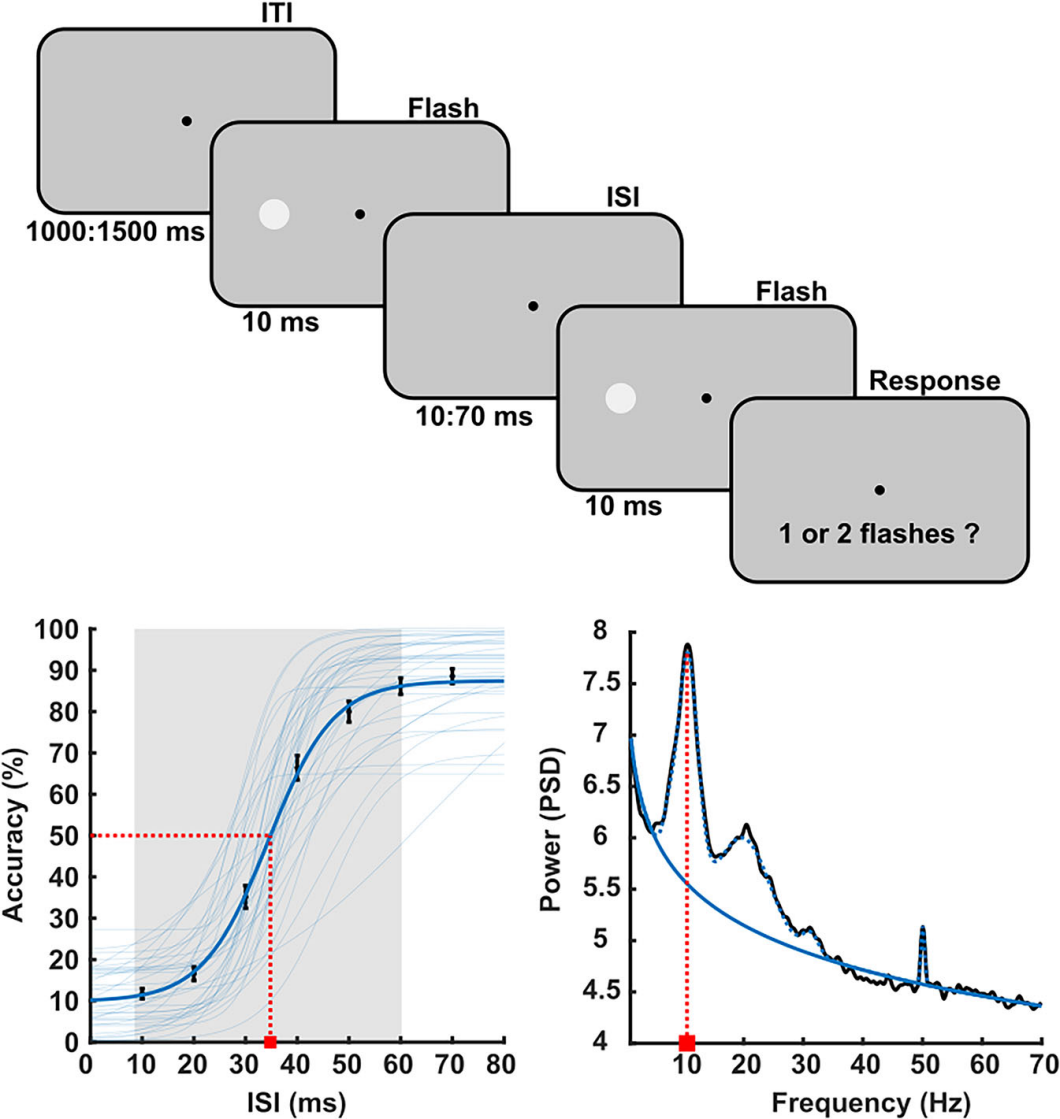
Experimental design and data modeling. Top, Illustration of the two-flash fusion task (see Materials and Methods). Bottom-left, Two-flash fusion accuracy is plotted against the ISI (average across participants). The thick blue line indicates the group average psychometric function fit; thin lines indicate single participants’ functions. The dotted red line indicates the threshold, and the shaded area indicates the function’ spread. Error bars indicate the standard error of the mean. Bottom-right, EEG spectrum of an example participant (channel Oz), where the solid blue line indicates the aperiodic fit, the dotted blue line indicates the periodic fit, and the dotted red line indicates the alpha frequency peak.

The experimental design is described in detail in Deodato and Melcher (2023). Briefly, at the beginning of the session, there was a resting-stage EEG block in which participants were asked to let their mind freely wonder and relax for 5 min with closed eyes (EC condition) and then with open eyes (EO condition). Next, participants were familiarized with the two-flash task, in which they were instructed to fixate on the center of the screen and report if they perceived one or two flashes. The main experiment trials began with a central fixation on a gray background lasting for 1,000–1,500 ms. This was followed by two consecutive flashes (∼20% contrast) presented on either the left or right side. Each flash lasted for 10 ms, and the interstimulus interval (ISI) was chosen from seven equally spaced values between 10 and 70 ms (Fig. 1). For each ISI, participants completed separately 10 training trials and 50 test trials. Trials were randomized and distributed in two blocks separated by a short break.

### Behavioral data analysis

The accuracy for each ISI was calculated by taking the average of the correct (i.e., two flashes) responses. A psychometric logistic function was applied to the accuracy data for each individual participant, and the fit of the model was determined using Cohen's pseudo-*R*^2^ and a nonparametric likelihood ratio test. Six participants were not included in the analysis since their lowest accuracy was above 30%, indicating that their responses were likely not due to temporal segregation (Boynton, 1972). One participant was excluded because the highest accuracy was 50%, and an additional five participants were removed due to poor model fit (<0.90), leaving 38 participants for further analyses (Deodato and Melcher, 2023).

### EEG analysis

EEG data were recorded from 32 scalp active electrodes (Fp1, Fp2, F7, F3, Fz, F4, F8, FC5, FC1, FC2, FC6, T7, C3, Cz, C4, T8, TP9, CP5, CP1, CP2, CP6, TP10, P7, P3, Pz, P4, P8, PO9, O1, Oz, O2, PO10) using the BrainVision Recorder software. EEG was acquired with a sampling rate of 1,000 Hz using the online reference at FCz. During preprocessing, an average of 0.5 bad channels per participant were detected via visual inspection and interpolated (range 0–6); independent component analysis was used to visually identify and remove an average of two components reflecting oculomotor artifacts. The reference was kept at FCz, and the average reference was avoided because broadband noise in a noisy channel would be distributed across all channels and affect the shape of 1/*f* activity.

To compute the EEG power spectral density (PSD), the resting state data were divided in 2 s segments with 50% overlap, and segments containing artifacts were rejected. The remaining segments were multiplied by a Hanning window, zero padded and Fourier transformed with a frequency resolution of 0.12 Hz, and converted to power. The aperiodic coefficient of the EEG was estimated with the “fitting oscillations and one over *F*” (FOOOF) algorithm, which fits a Lorentzian function and a sum of Gaussian peaks to the EEG spectrum (Donoghue et al., 2020). The exponent of the Lorentzian PSD function corresponds to the slope of the spectrum. Alpha power was estimated for each electrode and participant in the 8–12 Hz frequency range after removing the aperiodic activity (Deodato and Melcher, 2023). Spectral parametrization was obtained with default FOOOF peaks settings (width limits, [0.5 12]; minimum, 0; maximum, Inf; threshold, 2) over the 1–70 Hz frequency range to avoid oscillations and spectral plateau crossing the fitting range borders (Donoghue et al., 2020; Gerster et al., 2022).

### Statistical analysis

For each channel and resting-state condition, the correlation between the EEG aperiodic exponent and the psychometric slope of the behavioral data was calculated across participants using the Pearson's correlation coefficient. Notably, aperiodic exponents have been found to vary systematically with respect to age (Voytek et al., 2015) and alpha power (Muthukumaraswamy and Liley, 2018). Therefore, to control for the possible mediating effect of these variables, we conducted a mediation analysis (Baron and Kenny, 1986). First, aperiodic exponents and psychometric slopes were correlated with age and alpha power. Then, if a significant linear relationship was found, the regression between aperiodic exponents and psychometric slopes was recomputed while controlling for the effect of the intervening variable with multiple regression analysis (Baron and Kenny, 1986). Finally, to ensure that any result was not an artifact dependent on the parameters of the FOOOF spectral decomposition, we divided our sample in two groups (*n* = 19) based on the steepness of the psychometric slope (i.e., “shallow” vs “steep” group) and compared their spectra frequency-wise with a cluster-corrected independent samples *t* test.

Individual differences in spectral slopes have been linked to various domains of cognition and processing speed (Ouyang et al., 2020; Pathania et al., 2022; Pei et al., 2023; Euler et al., 2024). Thus, as a control, the same analyses were applied to test for a significant relationship between aperiodic exponents and two-flash fusion thresholds.

Statistical significance of the results was established with nonparametric methods and cluster-based multiple-comparison correction (10,000 permutations; Maris and Oostenveld, 2007). Although we had a priori hypotheses in terms of the direction of the effects, all statistical tests were two-sided. Data analyses were implemented with MATLAB custom scripts, the Fieldtrip Toolbox (Oostenveld et al., 2011), and the FOOOF-mat toolbox (Donoghue et al., 2020).

## Results

### Behavioral and spectral parametrization

**Figure 2.**
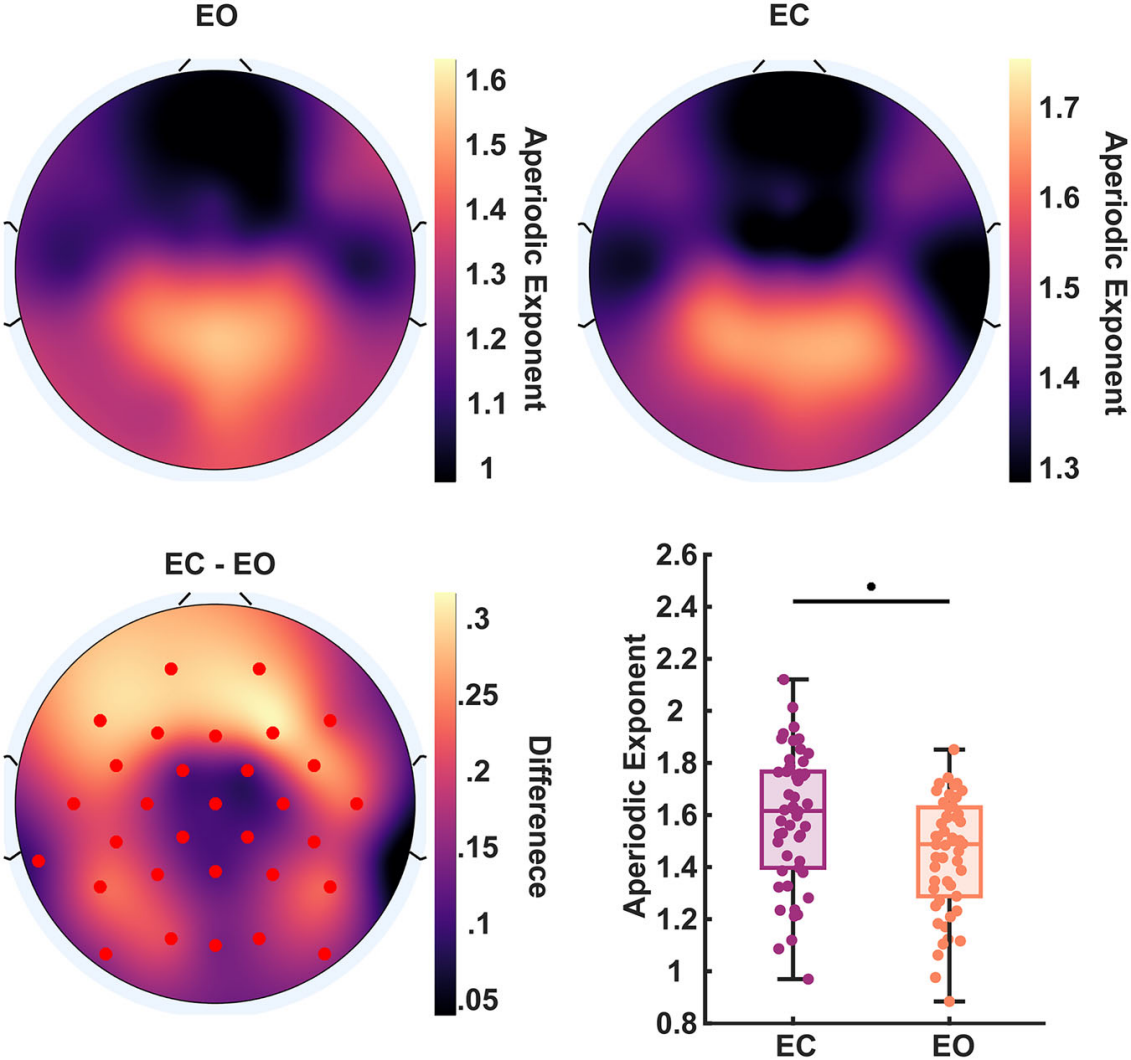
Aperiodic activity across the scalp. Top, Distribution of aperiodic exponents in the EO and EC resting state (averaged across participants) over the scalp. Bottom-left, Topographic map of the difference between the aperiodic exponents of EC and EO data; red markers indicate significant channels (*p* < 0.05). Bottom-right, Differences in the aperiodic exponent of the EO versus EC resting state at channel Oz.

In our sample (*n* = 38), the temporal discrimination threshold of the two-flash fusion task (mean, 35.2; SD, 6.57) was in line with previous research using similar paradigms to measure visual temporal resolution (Samaha and Postle, 2015; Deodato et al., 2024). Although the fit of the sigmoid model was good and significant for all participants included in the data analysis (Fig. 1; see Materials and Methods), the shape of the function varied greatly across participants. This variability is best indicated by the slope (mean, 0.21; SD, 0.102; range, 0.044–0.5) or spread (mean, 42.82; SD, 13.15; range, 15.4–60) distributions (i.e., the range between the lower and upper asymptotes of the function) and could reflect individual differences in internal noise (Yarrow et al., 2022; Deodato and Melcher, 2023). Resting-state EEG was recoded prior to the task, and the FOOOF (Donoghue et al., 2020) method was applied to achieve separation of periodic and aperiodic spectral components. This model provided excellent fits (mean *R*^2 ^= 0.994). Values of the EEG aperiodic exponent (i.e., the spectral slope) ranged between 1.07 and 1.61 across channels (mean, 1.35; SD = 0.15) and were greater over posterior scalp sites (Fig. 2), corroborating previous reports of EEG aperiodic activity (Dehghani et al., 2010; Donoghue et al., 2020). Notably, the aperiodic exponent was significantly smaller across the whole scalp in the EO condition with respect to the EC (*p* < 0.05, two-tailed, cluster corrected; Fig. 2).

### Age- and alpha power-dependent changes in aperiodic EEG

**Figure 3.**
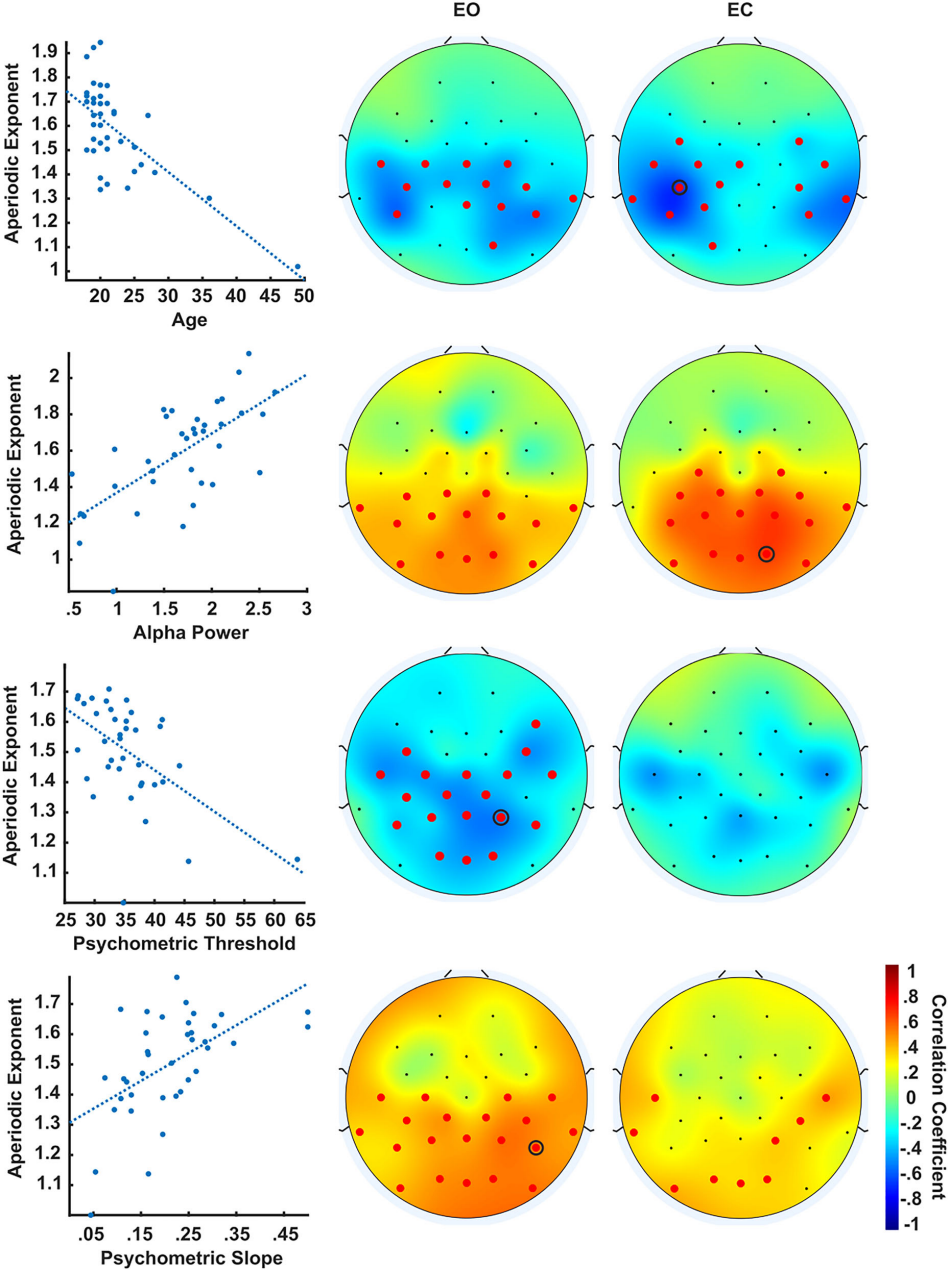
Correlation between two-flash fusion accuracy and resting state EEG. Each row shows brain–behavior correlations between aperiodic exponents and age, alpha power, psychometric threshold, and slope in the EO (middle) and EC (right) condition. The scatterplot on the left shows the data and regression line (dotted line) for the channel with the greatest (absolute) correlation coefficient (black circle in the topographic maps). The topographic maps reflect the correlation coefficients; red markers indicate significant channels (*p* < 0.05).

As expected, we found a negative correlation between age and aperiodic exponents over posterior and central electrodes (Fig. 3). Figure 3 shows the topographic distribution of the correlation coefficients for each correlation and the significant channels. For the sake of clarity and brevity, we report descriptive statistics (mean and range) of the significant correlation coefficients. The EEG of older participants was characterized by a flatter power spectrum in the EO (mean *r* = −0.42; range, −0.32 to −0.58; *p* < 0.02) as well as the EC (mean *r* = −0.43; range, −0.32 to −0.68; *p* < 0.007) conditions, replicating previous results concerning an age-dependent increase in neural noise (i.e., the neural noise hypothesis; Voytek et al., 2015). Aperiodic exponents were also positively correlated with alpha power across participants in the EO (mean *r* = 0.41; range, 0.33–0.53; *p* < 0.001) and EC (mean *r* = 0.52; range, 0.33–0.66; *p* < 0.001) conditions, reflecting a trade-off between synchronous and asynchronous brain activity in the posterior region of the scalp (Fig. 3). While evidence for this relationship has already been reported both between and within subjects (Muthukumaraswamy and Liley, 2018), its underlying mechanism remains debated (Voytek and Knight, 2015; Muthukumaraswamy and Liley, 2018; Evertz et al., 2022).

### Aperiodic EEG correlates with two-flash fusion slope

**Figure 4.**
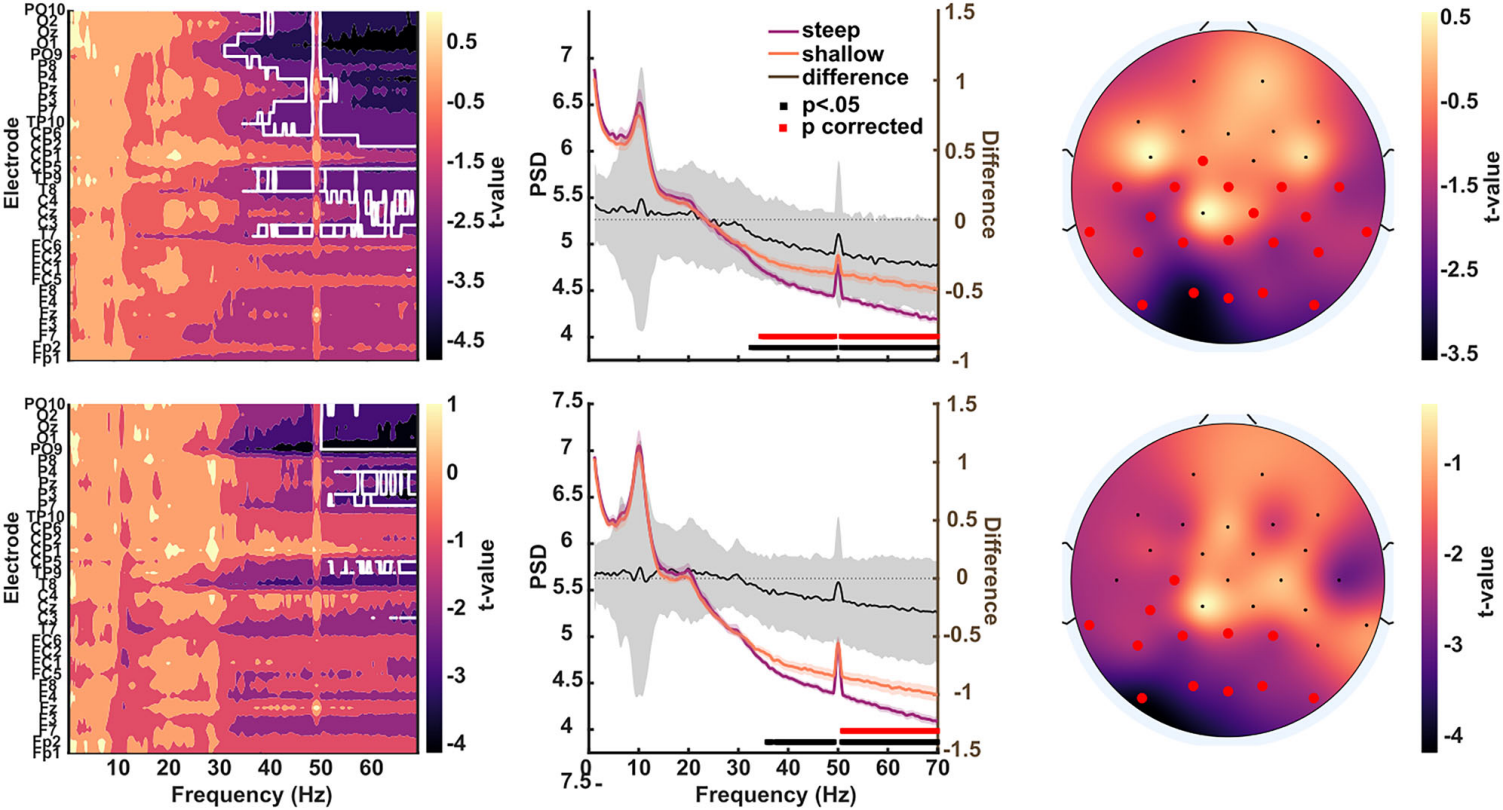
Spectral differences between participants with shallow and steep psychometric slopes. Left, Map of the *t* values across frequencies and electrodes; the white outline indicates significant differences (*p* < 0.05) after multiple-comparison correction. Middle, Comparison of the averaged EEG spectra of the two groups for channel Oz. Right, Topography of the *t* values averaged between 30 and 70 Hz. Red markers indicate electrodes with significant differences. The top row shows EO data; the bottom row shows EC data.

The main correlation analysis revealed a positive association between the psychometric slope of the two-flash fusion task and the aperiodic coefficient of the resting-state EEG (Fig. 3). Significant correlations emerged in the EC (mean *r* = 0.35; range, 0.32–0.44; *p* < 0.001) and EO (mean *r* = 0.43; range, 0.32–0.55; *p* < 0.02) condition and were localized on the posterior region of the scalp. We found no relationship between psychometric slopes and participants’ age or alpha power (all *p* > 0.05); the results survived a mediation analysis controlling for the effect of these intervening variables. Thus, this correlation likely reflects a direct relationship between aperiodic activity and two-flash psychometric curve slopes. Additionally, a frequency-wise spectral comparison between participants with steep and shallow psychometric slopes revealed a posterior cluster of significant broadband differences above 30 Hz in both conditions, consistent with a change in the aperiodic exponent (Fig. 4). Moreover, we found a smaller negative association between aperiodic exponents and two-flash fusion thresholds. This relationship was significant only in a cluster of posterior–central channels in the EO condition (mean *r* = −0.42; range, −0.33 to −0.52; *p* < 0.006) and did not survive the frequency-wise spectral comparison (all *p* > 0.05). Overall, these results indicate that participants with a flatter EEG spectrum had a shallower psychometric slope with respect to participants with greater aperiodic brain activity. In other words, greater aperiodic activity was associated with more variability in the two-flash integration/segregation task, consistent with an interpretation of neural noise.

## Discussion

We investigated the relationship between resting-state EEG and performance in a visual temporal acuity task to shed light on the role of aperiodic neural activity in the temporal resolution of visual sensory processing. We found that discrimination of the stimuli depended on broadband arrhythmic brain activity during the resting state. Specifically, shallower EEG spectral slopes predicted shallower psychometric functions for two-flash fusion across participants, independently of age and oscillatory power.

These findings have important implications for understanding sensory processing. Current proposals suggest that discrimination of rapid visual events (e.g., two flashes) relies on a mechanism of sensory sampling implemented by neural oscillations in the alpha frequency range (∼10 Hz; Samaha and Postle, 2015). Accordingly, using the same dataset, we have previously shown that two-flash fusion thresholds correlate with the speed of alpha oscillations (Deodato and Melcher, 2023). Here, we hypothesized that aperiodic activity, which reflects greater neural excitation (Gao et al., 2017), could impair the rhythmic inhibition delivered by alpha oscillations, thus affecting the temporal organization of incoming sensory information (Mazaheri and Jensen, 2010; Mathewson et al., 2011). In other words, the neural noise and unspecific excitation indexed by 1/*f* neural activity might be related to temporal precision in neural processing, causing inconsistencies in the integration/segregation of the same stimulus across different trials.

Previous research has linked these inconsistencies with the steepness of the two-flash fusion slope (Gruzelier and Venables, 1974; Deodato and Melcher, 2023). Generally, a shallow slope indicates poor reliability of the psychometric threshold estimate because it implies a larger interval of mixed responses and greater inconsistency in perceiving the same stimuli. This aligns with evidence that participants with shallow slopes do not exhibit a clear relationship between psychometric thresholds and neural oscillations (Deodato and Melcher, 2023) and are generally less susceptible to the effect of oscillatory phase on perception (Ten Oever et al., 2020). Conversely, a steep psychometric slope reflects consistent integration/segregation responses for each ISI. Notably, it has been suggested that the steepness of the psychometric function directly reflects the quality of sensory processing. This internal noise hypothesis (Schneider et al., 1989) posits that greater neural variability would impair consistent stimulus processing and cause shallower psychometric slopes in detection tasks (Allen and Nelles, 1996; Buss et al., 2006, 2009; Vilidaite and Baker, 2017; Sobon et al., 2019). In a similar vein, Yarrow and colleagues showed that the steepness of psychometric functions describing multisensory temporal integration is associated with the variability of modality-specific neural processing latencies (Yarrow et al., 2022).

The EEG spectral slope (i.e., the aperiodic exponent) has been recently interpreted as a measure of neural noise and information processing due to signal processing considerations as well as empirical findings. A flatter frequency spectrum indicates greater similarity of the signal to white noise, meaning that the EEG contains less information and autocorrelation, leading to a suboptimal state for neural communication (Hardstone et al., 2012; Voytek and Knight, 2015). Furthermore, this form of aperiodic noise has been suggested to be pivotal in some clinical conditions (Voytek and Knight, 2015; Pani et al., 2022) and to represent decreased signal-to-noise ratio in aging (Voytek et al., 2015; Tran et al., 2020). However, it should be noted that the relationship between aperiodic EEG, behavior, and cognition might not be so straightforward. Flatter EEG spectra have been linked with attentional deployment (Waschke et al., 2021) and greater working memory accuracy (Sheehan et al., 2018). Moreover, aperiodic features are heterogeneous across clinical conditions (Pani et al., 2022), and the absence of a relationship with cognitive performance has also been reported (Cesnaite et al., 2023).

Here, we propose that aperiodic activity and E:I balance, rather than being an unspecific index of neural noise, could be intrinsically related to oscillatory activity and the temporal organization of perception. Generally, one way the brain organizes information in time is through oscillations. Neural inhibition is critical for oscillatory dynamics to emerge (Freeman and Zhai, 2009) and orchestrate temporally sensitive computations (Ahissar and Arieli, 2001; Samaha and Postle, 2015). In the case of temporal integration of consecutive flashes, alpha oscillations provide a mechanism for sensory parsing by regularly pulsed inhibitory discharges. Specifically, periodic gating of neural activity allows the brain to segment incoming sensory information into discrete chunks, facilitating the seamless integration of successive stimuli into a coherent perceptual experience. In other words, these rhythmic pulses of inhibition create pauses between sensory events, allowing the brain to distinguish one from the next or integrate them, much like the spaces between words in a sentence help us understand the meaning of each word individually and in context. However, when neural excitation increases and induces neuronal firing independent of the phase of an oscillatory pacemaker, essentially acting as neural noise, this temporal organization mechanism might fail or become less reliable. A similar proposal has been advanced in the dynamic network communication framework: flattened power spectral slopes could result from increased background rates of neural firing caused by weak temporal coupling between neural spikes and the phase of oscillatory activity (Voytek and Knight, 2015). This explanation aligns with the observed trade-off between aperiodic activity and alpha power and further supports the finding that the desynchronized firing patterns commonly observed in flatter EEG spectra are an indicator of the balance between synaptic excitation and inhibition (Gao et al., 2017).

Furthermore, recent research suggested that individual differences in neural variability and aperiodic activity are reflected in the speed of neural processing (Garrett et al., 2013). Ouyang and colleagues, for example, found that the EEG power law exponent predicted individual differences in cognitive processing speed in an object recognition task (Ouyang et al., 2020). Additionally, age-related differences in cognitive tasks requiring speeded processing have been found to be mediated by aperiodic EEG exponents (Pathania et al., 2022). Similarly, steeper spectral slopes have been linked to better cognitive processing and faster reaction times (Euler et al., 2024). However, some evidence suggests that this link might hold only for cognitive control processes (Pei, 2023). On a related note, scale-free properties of EEG have been found to mirror those of detection of low-intensity flashes and sounds (Palva et al., 2013; Waschke et al., 2021) and the correlation structure of visual inputs (El Boustani et al., 2009), supporting a link with the variability, consistency, and temporal pace of neural processing. This variability might have clinical implications in both general and visual processing-related conditions. For example, Turri and colleagues recently reported that individuals affected by developmental dyslexia, a condition characterized by abnormal spatiotemporal organization of the visual flow, display flatter EEG spectral slopes compared with controls (Turri et al., 2023).

Finally, we found significant differences between aperiodic activity of the EO and EC conditions (Fig. 2), as well as qualitatively different scalp topographies for their brain–behavior correlations (Fig. 3). The widespread association between aperiodic EEG and two-flash fusion accuracy in the EO, rather than EC, condition could be explained in light of the similarity of the EO condition to the task condition. Similarly, while we cannot rule out the influence of ocular artifacts on aperiodic differences between EO and EC, these state-related changes could be a physiological excitatory response to sensory processing (El Boustani et al., 2009) and arousal (Lendner et al., 2020). However, the finding that the resting state in both conditions correlated with subsequent task performance suggests that aperiodic EEG exponents are a relatively stable trait within individuals. Indeed, aperiodic properties of individuals’ brain activity have been linked to stable, rather than momentary, cognitive performances (Euler et al., 2024) and considered a reliable biomarker for individual differences (Pathania et al., 2021) that is precise enough to discriminate single individuals in large EEG datasets (Demuru and Fraschini, 2020).

In conclusion, the present study offers a clear link between individual differences of resting-state aperiodic EEG (or neural E:I balance) and performance in a visual temporal discrimination task. We argue that heightened neural excitation could alter the rhythmic inhibition delivered by neural oscillations, causing inconsistent temporal segregation/integration of the visual stream. Overall, these results contribute to a growing literature highlighting the relevance of brain-related aperiodic activity in human cognition and behavior. As such, aperiodic activity should not be dismissed as a nuisance but considered as an important measure of neural noise and functional variability.

## Data Availability Statement

Data are publicly available in the Open Science Framework (osf.io/acb9n).
